# Dendritic cell phenotype and function in a 3D co-culture model of patient-derived metastatic colorectal cancer organoids

**DOI:** 10.3389/fimmu.2023.1105244

**Published:** 2023-01-25

**Authors:** Beatriz Subtil, Kirti K. Iyer, Dennis Poel, Lotte Bakkerus, Mark A. J. Gorris, Jorge Cuenca Escalona, Koen van den Dries, Alessandra Cambi, Henk M. W. Verheul, I. Jolanda M. de Vries, Daniele V. F. Tauriello

**Affiliations:** ^1^ Department of Tumor Immunology, Radboud Institute for Molecular Life Sciences, Radboud University Medical Center, Nijmegen, Netherlands; ^2^ Department of Cell Biology, Radboud Institute for Molecular Life Sciences, Radboud University Medical Center, Nijmegen, Netherlands; ^3^ Department of Medical Oncology, Radboud Institute for Molecular Life Sciences, Radboud University Medical Center, Nijmegen, Netherlands; ^4^ Oncode Institute, Nijmegen, Netherlands; ^5^ Department of Medical Oncology, Erasmus Medical Center, Rotterdam, Netherlands

**Keywords:** human dendritic cells, dendritic cell dysfunction, immunosuppression, 3D co-culture, tumor microenvironment, metastatic colorectal cancer, patient-derived tumor organoids

## Abstract

Colorectal cancer (CRC) remains one of the most aggressive and lethal cancers, with metastasis accounting for most deaths. As such, there is an unmet need for improved therapies for metastatic CRC (mCRC). Currently, the research focus is shifting towards the reciprocal interactions within the tumor microenvironment (TME), which prevent tumor clearance by the immune system. Dendritic cells (DCs) play a key role in the initiation and amplification of anti-tumor immune responses and in driving the clinical success of immunotherapies. Dissecting the interactions between DCs and CRC cells may open doors to identifying key mediators in tumor progression, and possible therapeutic targets. This requires representative, robust and versatile models and tools. Currently, there is a shortage of such *in vitro* systems to model the CRC TME and its tumor-immune cell interactions. Here we develop and establish a dynamic organotypic 3D co-culture system to recapitulate and untangle the interactions between DCs and patient-derived mCRC tumor organoids. To our knowledge, this is the first study investigating human DCs in co-culture with tumor organoids in a 3D, organotypic setting. This system reveals how mCRC organoids modulate and shape monocyte-derived DCs (MoDCs) behavior, phenotype, and function, within a collagen matrix, using techniques such as brightfield and fluorescence microscopy, flow cytometry, and fluorescence-activated cell sorting. Our 3D co-culture model shows high viability and extensive interaction between DCs and tumor organoids, and its structure resembles patient tissue sections. Furthermore, it is possible to retrieve DCs from the co-cultures and characterize their phenotypic and functional profile. In our study, the expression of activation markers in both mature and immature DCs and their ability to activate T cells were impacted by co-culture with tumor organoids. In the future, this direct co-culture platform can be adapted and exploited to study the CRC-DC interplay in more detail, enabling novel and broader insights into CRC-driven DC (dys)function.

## Graphical Abstract

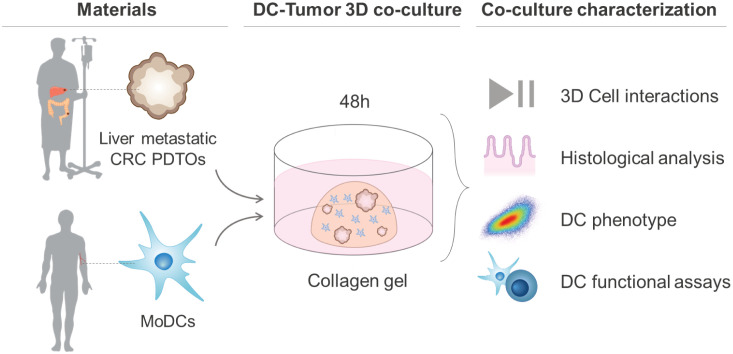

## 1 Introduction

Colorectal cancer (CRC) is one of the world’s most prevalent cancers and the second leading cause of cancer-associated mortality. For patients diagnosed at an early stage, the 5-year survival rate is as high as 90%. The survival rate, however, drops to only around 15% for advanced and metastatic disease (1). During disease progression, approximately half of patients develop metastases, with the liver as the most frequent metastatic site (1, 2). Most CRC-related deaths are thus not caused by the primary tumor but by distant metastases, which are often resistant to treatment (surgery, chemo-, targeted- and immunotherapy) (3). Treatment unresponsiveness in metastatic disease has been linked to a strongly immunosuppressive tumor microenvironment (TME) (4–6). CRC has evolved a number of escape mechanisms to induce immune suppression including the exclusion and corruption of immune cells. This either occurs through direct cell-to-cell contact or paracrine signaling, for which the molecular mechanisms remain incompletely understood. It is therefore crucial to further our understanding of the complex interactions between tumors and immune cells in order to eventually develop and improve treatment strategies.

Dendritic cells (DCs) are the key orchestrators of anti-tumor immune responses and have been shown to be locally and systemically impaired in cancer patients, including CRC (7). Under physiological conditions, DCs constantly patrol and scan the environment for danger signals in an immature state. In the presence of tumor antigens and danger signals, DCs become activated, mature, and trigger anti-tumor immune responses (8). As such, they have the unique capacity to link the innate and adaptive immune system by (cross)-presenting antigens and priming T cells (9, 10). Importantly, this implies that effector T cell responses against cancer-specific antigens require functional and mature DCs (11, 12). In an immunosuppressive tumor setting, DCs become locked in or regress into an immature state, which compromises their ability to activate T cells leading to T cell anergy and Treg recruitment, and in this way foster tumor tolerance (13–16). In agreement, several studies implicate dysfunctional DCs in immune evasion, tumor growth, metastasis initiation, and treatment resistance in CRC, clearly indicating that DCs not only dictate the outcome of anti-tumor immunity but also of treatment response (17).

Despite the critical role of DCs in anti-tumor immunity and immunotherapy response in cancer, the CRC-specific mechanisms shaping and regulating DC phenotype and functionality are still largely unknown. Unveiling the crosstalk between CRC and DCs brings hope for identifying and modulating key mechanisms and pathways involved in tumor progression and spread. This presupposes unexplored opportunities for therapeutically reverting tumor-induced DC suppression and enhancing anti-tumor immunity (16, 18).

To gain insights into these CRC-DC interactions, it is crucial to have suitable and representative models and tools to study and unveil the underlying molecular mechanisms. Animal models of CRC, despite mimicking several features of the human disease, cannot faithfully represent the complexity of the human TME. In addition, mouse and human DC subsets differ considerably, impairing subset-specific studies. There is a shortage of *in vitro* systems that faithfully model the primary and metastatic CRC TME and its tumor-stromal cell interactions. Moreover, existing *in vitro* 2D co-culture systems, albeit highly accessible and inexpensive, fail to recapitulate cell shape and polarization, as well as, tumor complexity, heterogeneity, and 3D spatial interactions within the TME (19). As such, there is a pressing need for clinically and physiologically relevant systems that enable the dissection of cellular interactions, and assessment of individual contributions to certain phenotypes within the CRC TME.

3D co-culture models using tumor spheroids or organoids aim to overcome the shortcomings of previous *in vitro* models and shorten the gap between the ability to manipulate the system and physiological relevance (20, 21). These appear to be promising tools to better recreate the complex interactions between tumor cells and other cells that compose the TME, including DCs. To date, spheroid-based TME models for different tumors have been developed to investigate DCs behavior and plasticity (22–24). However, spheroids fall short of representing glandular differentiation and polarization, as well as tumor heterogeneity, one of the key features of CRC. In contrast, patient-derived tumor organoids (PDTOs) are heterogenous self-organizing populations of tumor cells, resembling the architecture, and preserving morphological and mutational features of the tissue of origin (20, 21, 25–27). As such, PDTOs represent a relevant and appropriate *in vitro* platform for biological studies and testing personalized medicine. Furthermore, studies have shown that organoid co-culture systems can be successfully used to study and recapitulate interactions between cancer cells and immune cells (27–30). Recently, a co-culture model of MoDCs and healthy human gastric organoids in a controlled and complex microphysiological chip platform was developed (31). Yet, studying DC-CRC interactions using PDTOS in a 3D context is still an unexplored field with many opportunities.

Here, we describe the development of a representative and relevant 3D co-culture system between human DCs and patient-derived liver metastatic CRC organoids. In our co-culture system, monocyte-derived DCs (MoDCs) from healthy donors were used as a human DC model and cultured in a 3D collagen matrix in the presence or absence of PDTOs. MoDCs were added to the co-culture system in an immature (iDCs) or mature state (mDCs) as both can be found infiltrating the TME, and have different roles and behaviors (12). The two CRC liver metastasis PDTOs used in this study were selected based on their different morphology – cystic and compact/dense. Furthermore, we present an associated toolbox that includes live-cell microscopy, histological analysis, immunofluorescence, flow cytometry, and cell sorting to assess and characterize the tumor effect on the behavior, phenotype, and functionality of DCs.

## 2 Materials and methods

### 2.1 Human samples and patient material

Blood samples (buffy coats) from healthy donors were obtained *via* Sanquin Blood Bank (Sanquin Bloedvoorziening, Nijmegen, the Netherlands). Needle biopsies and resection material from liver metastasis of CRC patients and tumor tissue sections were obtained within the context of the ORCHESTRA trial (NCT01792934). All healthy donors and patients gave written informed consent.

### 2.2 Isolation of peripheral blood mononuclear cells

Peripheral blood mononuclear cells (PBMCs) were isolated from buffy coats by density gradient centrifugation at 500 x g at room temperature (RT) for 30 minutes using Lymphoprep medium (StemCell Technologies, 07861). The layer containing the PBMCs was isolated and extensively washed with PBS supplemented with 0.1% BSA and 2 mM EDTA. The residual red blood cells were removed using red blood cell ACK lysis buffer (Gibco, A1049201). Monocytes and pan T cells were isolated from the healthy donor PBMCs as described below.

### 2.3 Isolation and differentiation of (mature and immature) monocyte-derived dendritic cells

Monocytes were isolated from the PBMCs using CD14 Microbeads (Miltenyi Biotec, 130-050-201) according to the manufacturer’s instructions. For differentiation of MoDCs, monocytes were cultured in X-VIVO 15 (Lonza, BE02-060F) supplemented with 2% human serum and with 450 U/ml GM-CSF (Miltenyi Biotec, 130-093-868) and 300 U/ml IL-4 (Miltenyi Biotec, 130-093-924) for differentiation for 5 days (cytokines and medium were refreshed at day 3). After differentiation, MoDCs were matured for 24h with 1000 U/ml IL-6 (Proteintech, HZ-1019), 1000 U/ml IL-1β (Peprotech, 200-01B), 500 U/ml TNF-α (Peprotech, 300-01A), and 10 µg/ml PGE2 (Prostin E2 Pfizer). On day 6, immature (iDCs) and mature (mDCs) MoDCs were harvested after 1h incubation at 4°C in cold PBS and with the help of a cell scraper.

### 2.4 Isolation of pan T cells

For the allogeneic T cell assays/mixed lymphocyte reaction assays, T cells were isolated from the PBL fraction of PBMCs using the Pan T cell isolation Kit *(*Miltenyi Biotec, 130-096-535*)* according to manufacturer instructions.

### 2.5 Establishment of patient-derived tumor organoids

The following procedure was used to establish patient-derived tumor organoids [Iyer, Poel, et al. (manuscript in preparation)]. Patient biopsies were collected in Advanced DMEM/F12 (Gibco, 12634010), supplemented with 1x GlutaMAX™-I (Gibco, 35050038), 10 mM HEPES Buffer solution (Gibco, 15630056), and Penicillin Streptomycin (10000 U/mL Penicillin, 10000 µg/mL Streptomycin) (hereafter referred to as +3 Advanced medium), and 10 μM Rho-kinase inhibitor. The collection medium was removed, and the biopsies were washed with cold HBSS (Lonza). Then, the biopsies were transferred to a petri dish for mechanical digestion, washed twice with +3 advanced medium, and transferred into a 15 ml tube. The tumor tissue was incubated in 20 mg/mL Collagenase-II (Sigma-Aldrich) and 10 μM Rho-kinase inhibitor for tissue dissociation, and placed in the water bath at 37°C for 30 minutes. After tissue digestion, 10% FBS (Gibco) was added to stop the collagenase digestion. The minced and digested tissues were passed through a pre-wetted 200μM filter (Pluriselect) into a 15 ml tube. The tissue was centrifuged for 5 minutes at 400 x *g* and 4°C. Lastly, the supernatant was removed, and the pellet was resuspended in Cultrex Ready Basement membrane extract (BME) (Bio-Techne, 3434-050-RTU).

### 2.6 Culture of patient-derived tumor organoids and single cell counting

Organoids were cultured in 25 µl domes of 70% (v/v) BME in +3 Advanced medium supplemented with 5% (v/v) R-spondin-CM (provided by courtesy of the Kuo lab, Stanford University), 5% (v/v) Noggin-CM (provided by courtesy of the Clevers lab, Hubrecht Institute), B27 Supplement without vit. A (Gibco, 12587010), 10mM Nicotinamide (Sigma, N0636), 0.2 mg/mL Normocin™ (*Invivo*Gen, ant-nr-1), 1.25 mM n-acetylcysteine, 10 nM Gastrin-I (human) (Bio-Techne, 3006/1), 50 ng/mL hrEGF (Peprotech, AF-100-15), 3 µM SB202190 (Seleck, S1077), and 2 µM Galunisertib (LY2157299) 26. Organoids were kept until passage 25. For passaging and co-cultures, PDTOs were collected by adding ice-cold medium to dissolve the BME. For the co-culture, a trypsinization step was included to count the number of PDTO single cells present in a certain volume. In this study two PDTOs were used and selected based on their different morphology: PDTO013 hereafter referred to as PDTO cystic and PDTO024 as PDTO dense. [Iyer, Poel, et al. (manuscript in preparation)].

### 2.7 Generation of co-cultures between PDTOs and DCs in a 3D collagen gel

Bovine Collagen type I (fibrillar), the most widely used and investigated extracellular matrix for 3D cell culture, was used as a scaffold for PDTOs and DCs co-cultures. The collagen mix consisted of 3.1 mg/ml Bovine PureCol I (Advanced Biomatrix, 5005) (final concentration of 1.7 mg/ml), 10x MEM (Gibco, 11430-030) (final concentration of 0.74x), 7.5% sodium bicarbonate (Gibco, 25080-060) (final concentration of 0.28%), and the cells in X-VIVO 2% human serum. The mixture was prepared as described elsewhere (32). To avoid fragmentation by mechanical disruption, the PDTOs were collected carefully in a volume corresponding to the desired amount of counted cells in another identical sample. PDTOs and/or DCs were embedded in the collagen mix in a ratio of 1:1 (50,000:50,000 cells per 25 µl dome). The collagen gel domes were solidified for 30-45 min at 37°C, inverted to ensure polymerization in 3D and prevent cell attachment to the bottom of the well. The gels were kept in culture for 48h in X-VIVO 15 + 2% human serum.

### 2.8 Cell labeling for live imaging and flow cytometry

The cell viability within the 3D collagen gels was assessed using the ReadyProbes^®^ Cell Viability Imaging Kit (ThermoFisher, R37609): NucBlue™ Live reagent stains the nuclei of all the cells and NucGreen™ Dead reagent stains only the nuclei of cells with compromised plasma membranes. The viability was quantified in the conditions with mDCs and iDCs alone, by using three different images of each condition from two different experiments/donors. The percentage of viable cells was calculated based on the cells stained with NucBlue™ Live and NucGreen™ Dead (cells stained with NucBlue™ – live cells, NucBlue™ and NucGreen™ – dead cells). Ibidi µ-Plate 24 Well Black ID 14 mm (82426) were used for imaging. For live imaging, the microscope Zeiss Axio Observer with a 10x magnification was used. For the time series, images were taken every 30 seconds or 1 minute. Images and movies were processed using Image J (Fiji). For the flow cytometry-based phagocytosis/uptake assay, prior to the generation of the co-cultures, DCs were stained with a CFSE cell-labeling dye (C34554, Invitrogen) and PDOs with a FarRed cell-labeling dye (C34564, Invitrogen) according to manufactures’ instructions.

### 2.9 Co-culture fixation, embedding, and slide preparation

Co-cultures were fixed in formalin for 1h to preserve the co-culture structure, cell morphology, and localization. The fixed co-cultures were placed in Tissue-Tek^®^ Paraform^®^ cassettes (Sakura, 7019) and embedded in paraffin. The formalin-fixed, paraffin-embedded (FFPE) co-cultures were sectioned at 5 µm thickness with a microtome (Microm) for stainings and mounted on SuperFrost microscope slides (VWR, 631-9483). For 3D immunofluorescence stainings, the co-cultures were fixed for 1h with 4% PFA.

### 2.10 Immunofluorescence in collagen gels and slides

The following protocol was performed for immunofluorescence stainings in paraffine sections: following deparaffinization and rehydration, the slides were boiled in Tris-EDTA buffer for antigen retrieval. After incubation with blocking solution, the primary antibodies were added: 1:300 anti-CD11c (Abcam, ab52632) and 1:100 anti-pan cytokeratin (PanCK) (Abcam, ab7753) and incubated overnight at 4°C in a humidified chamber. The following day, the slides were washed three times with PBS. Slides were incubated for 1h at room temperature in the dark with 2.5 μg/ml DAPI (Roche, 10236276001), and the secondary antibodies, donkey anti-rabbit 488 (Invitrogen, A21206) and donkey anti-mouse 647 (Invitrogen, A31571) both 1:200. Samples and slides were washed and mounted with Fluoromount Mounting Medium (Sigma-Aldrich, F4680).

A slightly different protocol was followed for immunofluorescence stainings in 3D collagen co-cultures. Cultures were incubated with a blocking solution (20 mM Glycine, 2% BSA, and 0.3% Triton in Phosphate buffer) for 1h at RT. Primary antibodies were added as described above for the paraffin sections. After overnight incubation and PBS washes, 2.5 μg/ml DAPI and the secondary antibodies were added and incubated for 2h at RT. Following the washing steps, the cultures were mounted in a microscopy slide with Mowiol (Sigma-Aldrich, 81381).

Once dry, the slides were imaged with a Zeiss AI Sample Finder microscope or with a Zeiss confocal laser scanning microscope LSM880. Image processing and analysis were performed using Image J (FijiJ). To quantify DCs location in relation to the tumor border, images were processed and segmented into regions of interest. Two distance maps were applied (normal and inverted) generating positive and negative values for each DC location.

### 2.11 Multiplex immunohistochemistry of patient samples

Multiplex immunohistochemistry of patient FFPE samples was done in sequential staining cycles using the Opal 7-color Automation IHC Kit (Akoya Biosciences, NEL801001KT) on the BOND RX IHC & ISH Research Platform (Leica Biosystems), which was optimized and performed as described before (33, 34). The multiplex panel consisted of 1:200 anti-CD14 (Cell Marque, 114R-16) with Opal620, 1:200 anti-CD19 (Abcam, ab134114) with Opal690, 1:150 anti-BDCA2 (Dendritics, DDX0043) with Opal540, 1:100 anti-CD1c (Thermo Fisher Scientific, TA505411) with Opal520, 1:100 XCR1 (Cell Signaling Technologies, 44665S) with Opal570 and 1:1500 anti-pan cytokeratin (Abcam, ab86734) with Opal650. Slides were counterstained with DAPI for 5 minutes and enclosed in Fluoromount-G mounting medium (SouthernBiotech, 0100-01). Whole tissue slides were imaged using the microscope Vectra 3 Automated Quantitative Pathology Imaging System (Version 3.0.4, PerkinElmer Inc.). For comparison to the co-cultures with PDTOs, only DAPI, CD1c, and Pan cytokeratin are shown.

### 2.12 Hematoxylin and Eosin staining

Hematoxylin-Eosin (HE) histological stainings were performed according to standard protocols. Once dry, the slides were imaged with a slide scanner (3DHISCTECH Pannoramic 1000, Sysmex).

### 2.13 Co-culture dissociation/disaggregation

After 48h of co-culture, Collagenase I (Sigma-Aldrich, C0130) solution was added to the co-culture medium (20 U/ml) for collagen dissolution and co-culture disaggregation for 45 min at 37°C. The cells were collected and viability was assessed prior to centrifugation using trypan blue and BIO-RAD TC20™ Automated Cell Counter. The cells were washed and used for flow cytometry or sorting staining protocols. Samples containing PDTOs were filtered through a Corning^®^ Cell Strainer (70 µm Nylon MESH) before the staining protocol. For the flow cytometry-based phagocytosis/uptake assay a trypsinization step was included to yield single cells before acquisition.

### 2.14 Flow cytometry

Phenotypic characterization of DCs surface markers was performed as followed: Firstly, Fc receptors were blocked using Fc blocking reagent (Miltenyi, 130-059-901) for 10 min at 4°C to avoid non-specific antibody binding. Secondly, cells were stained with fixable Viability Dye eFluor™ 506 (Invitrogen, 65-0866-14) for 20 min at 4°C. Thirdly, cells were stained with directly labeled primary antibodies - anti-CD86-PE (BD Biosciences, 555658) 1:15, BV421 anti-PD-L1-BV421 (BD Biosciences, 563738) 1:25, anti-HLA-DR-PerCP (BioLegend, 307628) 1:20 - for 25 min at 4°C. Lastly, cells were washed before acquisition. The acquisition was performed on a FACSVerse flow cytometer (BD Biosciences). The acquired data was analyzed with FlowJo Version 10. The values were plotted as mean fluorescence intensity (MFI), mean ± standard deviation (SD), normalized to the conditions with only DCs (iDCs or mDCs, correspondingly). Relevant gating strategies used are depicted in the Results section.

### 2.15 Fluorescent-activated cell sorting

To isolate a pure population of DCs, for functional readouts, fluorescent-activated cell sorting (FACS) was performed using the BD FACSMelody Cell Sorter. To sort out residual PDTOs after the filtration step, the sterile antibody anti-CD326 (EpCAM)-PE human (Invitrogen, 12-9326-42) at 1:30 dilution was used. Cells were sorted with >98% purity. Relevant gating strategies are depicted in the Results section. Sorted DCs were then plated in triplicates with T cells for a mixed lymphocyte reaction as described in the following section.

### 2.16 Mixed lymphocyte reaction

Allogeneic T cell assays (mixed lymphocyte reaction) were performed to evaluate the ability of DCs to induce T cell proliferation after being sorted from the co-cultures. To detect T cell proliferation Pan T cells were labeled with 5 µM of Cell Trace CFSE (Invitrogen, C34554). DCs and CFSE-labelled T cells were seeded in a round-bottom 96-well plate at a 1:10 ratio in triplicates and co-cultured for 6 days. In order to assess T cell proliferation, at day 6, T cells were collected and stained with anti-CD8-APC (BD Biosciences, 555369) at 1:50 for 25 min at 4°C. Samples were acquired and analyzed with FlowJo Version 10. The values were plotted as mean MFI of the average of technical replicates, normalized to the conditions with DCs only (iDCs or mDCs), mean ± SD. Relevant gating strategies used are depicted in the Results section.

### 2.17 Statistical analysis

All statistical analyses were performed using GraphPad Prism V9 (GraphPad Software Inc, San Diego, CA). Unless otherwise indicated, results are presented as mean ± SD in scattered dot plots. Concerning MFI values, the statistical significance between different conditions was analyzed by a mixed-effects model followed by a Dunnett’s *post-hoc* multiple comparisons test on the log2 transformed ratio values. When comparing DCs distribution in the tissue an unpaired t-test was used. The statistical significance was annotated as follows: *p < 0.05, **p < 0.01, ***p < 0.001, ****p < 0.0001.

## 3 Results

### 3.1 DCs remain viable in 3D during co-culture with CRC liver metastasis PDTOs

**Figure 1:**
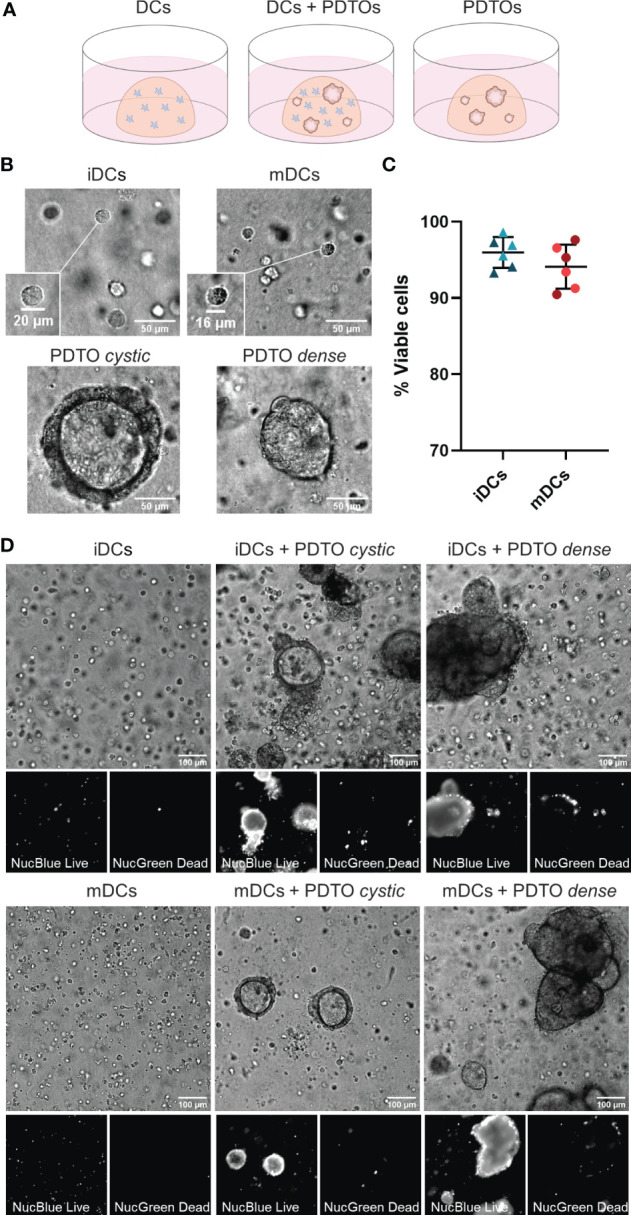
3D co-culture of PDTOs and DCs: Setup, morphology, and viability. **(A)** Schematic representation of the co-culture system and the three different conditions used in this study: DCs cultured alone, DCs and PDTOs co-cultured, and PDTOs cultured alone. **(B)** Closeup and morphology of iDCs and mDCs. The PDTOs are derived from CRC liver metastasis of two different patients, PDTO cystic as the name suggests presents a cystic morphology, whereas PDTO dense has a compact morphology. **(C)** Quantification of mDCs and iDCs viability when cultured alone in 3D in the collagen matrix after 48h, based on NucBlue™ Live and NucGreen™ Dead stainings, in two different experiments/donors. **(D)** The viability of mDCs, iDCs, and PDTOs was evaluated, after 48h of co-culture, during live imaging with NucBlue™ Live reagent (staining the nuclei of all cells) and NucGreen™ Dead reagent (staining only dead cells). The large majority of DCs seem to be viable alone, and in co-culture. iDCs - immature MoDCs, mDCs - mature MoDCs, PDTOs - patient-derived tumor organoids.

The main goal of this study was to set up a robust and dynamic 3D co-culture system between human DCs and CRC liver metastasis PDTOs that would allow investigation of how patient tumor cells shape DCs behavior, phenotype, and function. For this system, immature and mature DCs were cultured in a 3D fibrillar collagen drop in the presence or absence of PDTOs (**Figure 1A**). We observed that both DCs and organoids remained in the 3D collagen matrix and did not attach to the bottom. Within our co-culture system, it was also possible to visualize size differences between iDCs and mDCs, with the latter being slightly smaller, and between PDTO cystic and PDTO dense (**Figure 1B** and **Supplementary Figure 1**). During live cell imaging, NucBlue™ Live (staining all cells) and NucGreen™ Dead (staining dead cells) reagents were used to evaluate the viability of cells in the collagen matrix. After 48h the majority of DCs were alive both alone - mean viability of iDCs and mDCs cultured in the absence of PDTOs was 97 and 95%, respectively - and in co-culture with PDTOs (**Figure 1C, D**).

### 3.2 DCs interact with CRC liver metastasis PDTOs and engulf tumor-derived fragments in the 3D co-culture system

**Figure 2:**
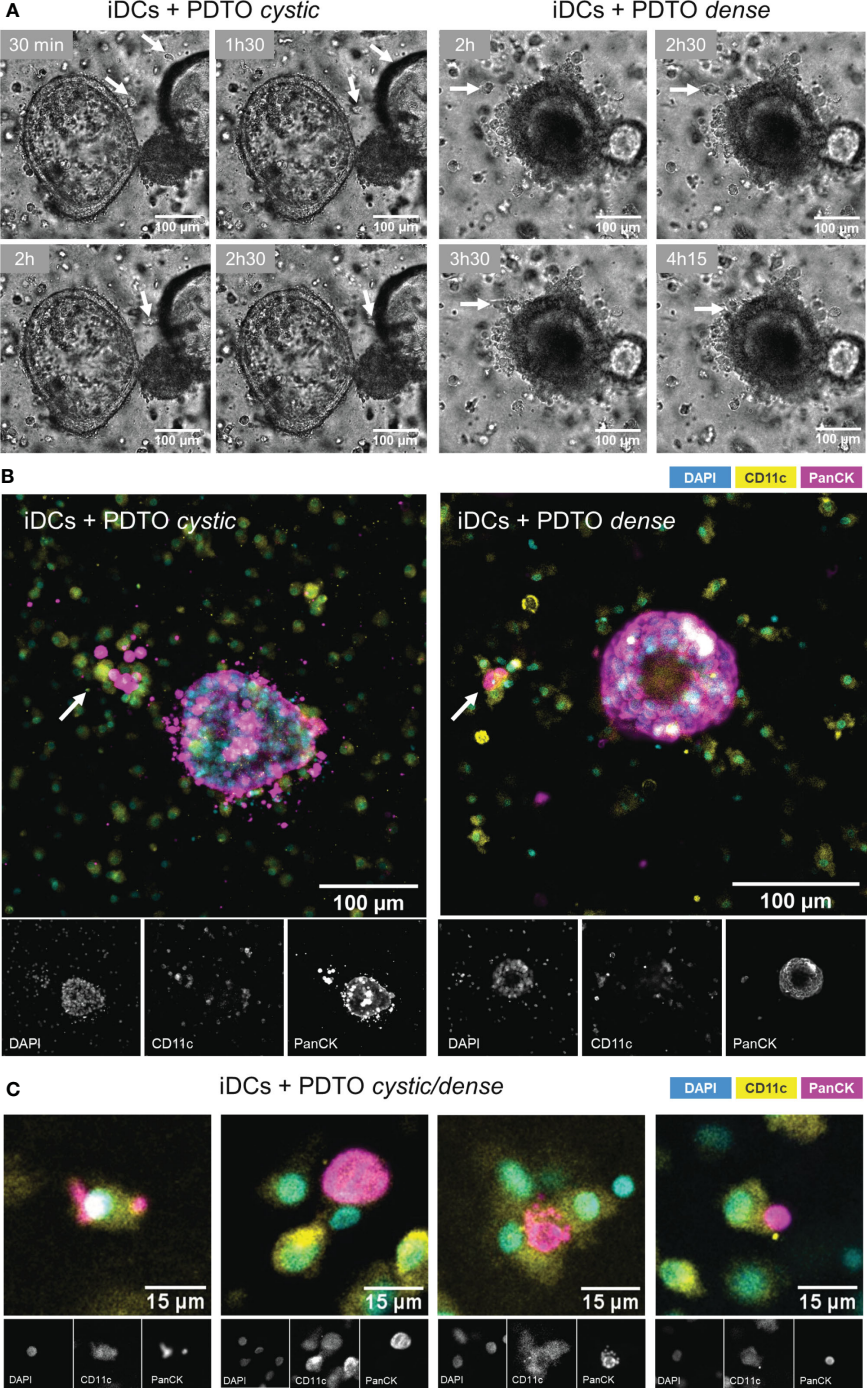
Visualization of DCs - PDTOs interactions within the co-culture. **(A)** Time series frames: iDCs establish direct contacts with co-cultured PDTOs by migrating towards and agglomerating near the tumor organoid borders (examples pinpointed by the arrows). **(B)** 3D immunofluorescence stainings with DAPI, CD11c, and PanCK, to distinguish DCs and tumor cells. **(C)** Examples of iDCs in close proximity to and surrounding/engulfing tumor-derived fragments. iDCs - immature MoDCs, mDCs - mature MoDCs, PDTOs - patient-derived tumor organoids.

To confirm that the DCs were not only alive but also actively interacted with tumor cells, we recorded time series. As shown in **Figure 2A** (**Supplementary Movie 1** and **2**), during co-culture iDCs dynamically interacted with the tumor organoids by migrating towards and into the organoid, as well as, agglomerating near and engaging with the border. Next, we performed 3D immunofluorescence in fixed samples at 48h. CD11c and PanCK stainings allowed clear differentiation of DCs and tumor cells, respectively. Stainings also confirmed the presence of iDCs in close proximity to and gathered around and inside the tumor organoids (**Figure 2B**). Additionally, it is possible to observe iDCs surrounding and seemingly engulfing tumor cells or -derived fragments (**Figures 2B, C**). An additional assay, flow cytometry-based, was performed by labeling DCs and PDOs with fluorescent dyes, which provides further evidence for direct and functional interaction between iDCs and tumor cells, and engulfment/uptake of tumor cells within the co-culture (**Supplementary Figure 2**). In our system iDCs were found to be more frequently interacting with and taking up tumor cells than mDCs. Together, these results indicate that our co-culture system supports DC viability and function, and facilitates DC-tumor interactions, including the uptake of tumor cells or cell-derived fragments.

### 3.3 DCs distribution in relation to PDTOs in the 3D co-culture system is maturation status-dependent and resembles patient tumor samples

**Figure 3:**
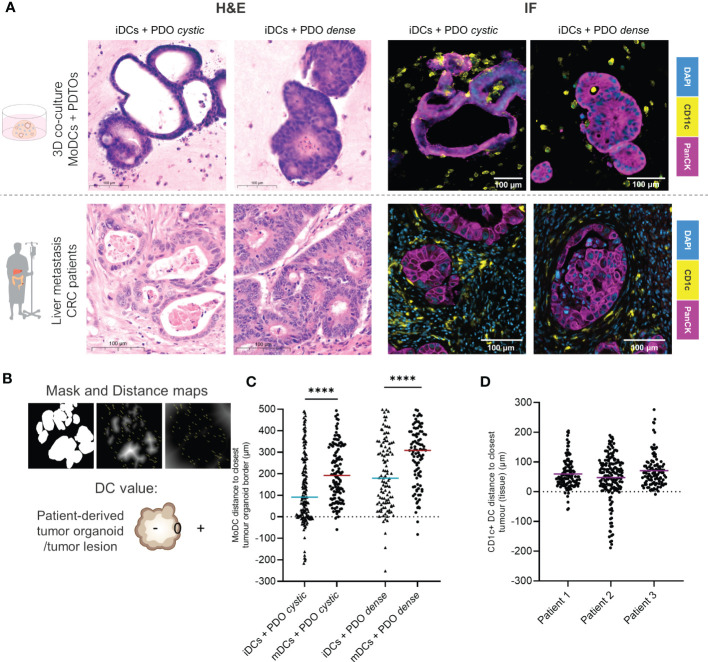
DCs distribution in relation to the tumor lesions within the co-culture in comparison to patient tumor samples by immunofluorescence. **(A)** Parallel between H&E and immunofluorescence stainings of DCs and organoids/tumors in fixed sections of the 3D co-culture and of CRC liver metastasis in patients. On the lF panel, representative examples of immunofluorescence stainings of CD11c and PanCK in the co-culture and of CD1c and PanCK in liver metastasis tumor sections. DCs are present agglomerating around, surrounding and infiltrating tumor organoids and tumor lesions in patients. Additional and larger images are included in **Supplementary Figures 3** and **4**. **(B)** Analysis of DCs distribution within the co-culture by image processing including segmentation and distance maps (normal and inverted). Each DC was assigned a positive, 0 or negative value depending on whether they were found outside, at the border, or inside the tumor, respectively. **(C)** The scatter dot plot shows differences in DC distribution around and inside the tumor organoids. Each dot represents one DC, line at the median. The p values were determined using an unpaired t-test. Statistical significance was annotated as follows: ****p < 0.0001 based on two sections from two independent experiments. **(D)** The scatter dot plot shows differences in DC distribution around and inside the tumor lesions, based on sections from 3 different patients. Each dot represents one DC, line at the median. The p values were determined using an unpaired t-test. Statistical significance was annotated as follows: ***p < 0.001, ****p < 0.0001 based on sections from 3 different patients. iDCs - immature MoDCs, mDCs - mature MoDCs, PDTOs - patient-derived tumor organoids.

To evaluate the co-culture structure and architecture we subjected our co-culture system to a standard H&E staining. We observed that DCs are distributed evenly throughout the matrix when alone in the collagen matrix (**Supplementary Figure 3A, B**). In the presence of the tumor organoids, iDCs can be found surrounding the tumor cells (**Supplementary Figure 3C**). Histological comparison of H&E staining in tissue sections obtained from two different patients with CRC liver metastasis and co-culture sections confirmed that the two organoid morphologies, PDTO cystic and PDTO dense, mimic two types of tumor lesions present in patients (**Figure 3A** and **Supplementary Figure 3D**).

We next looked in more detail at the distribution of DCs, relative to tumor organoids. Immunofluorescence of CD11c (MoDCs) and PanCK (tumor cells) was performed in fixed sections of the co-cultures after 48h. Comparison to patient tissue sections, stained with CD1c—indicating DCs with a myeloid origin—and PanCK, suggested that our co-cultures achieve representative interactions between cancer cells and DCs, even in the absence of other stromal cells (**Figure 3A**). DCs, in particular iDCs, are found close to the tumor organoid border and inside, with a satellite-like disposition around tumor organoids and small clusters of tumor cells (**Figure 3A** and **Supplementary Figures 4A, B**). Therefore, we hypothesized that DCs distribution within the co-cultures may be influenced by their maturation status.

To assess DCs distribution in relation to tumor glands in both the co-culture and patient tissue sections, distance maps were applied generating positive or negative values for each DC location depending on their distance to the closest epithelial tumor gland (**Figure 3B**). The results show that iDCs are significantly in closer proximity to the tumor border and are more often found inside the tumor when compared to mDCs for both PDTOs. With this analysis, we demonstrate that DC distribution within the co-culture system differs depending on DC maturation status. Moreover, the results suggest that both iDCs and mDCs are found in closer proximity to PDTO cystic than to PDTO dense (**Figure 3C**). In the analyzed patient sections of CRC liver metastasis, we observed that CD1c+ DCs agglomerate close to tumors (<100 µm) surrounding and infiltrating lesions, as seen in our co-culture system (**Figure 3D**). The distribution and position of myeloid DCs (CD1c+ DCs) were found to be comparable to the distribution of iDCs within the co-culture in terms of range and mean distance to the tumors.

### 3.4 Phenotypic characterization of DCs after retrieval from the 3D co-culture

**Figure 4:**
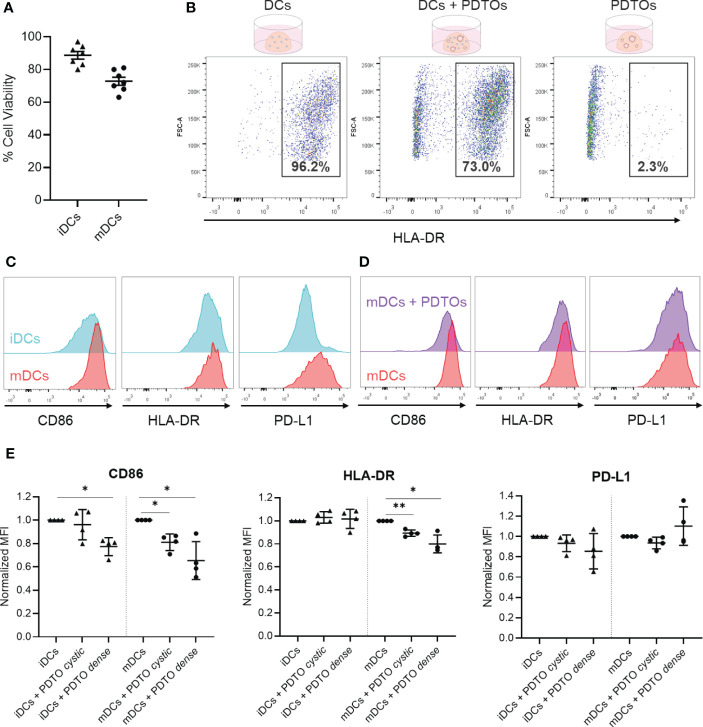
Recovery of DCs after co-culture with tumor PDTOs - viability, gating strategy, and phenotypic characterization to assess tumor-induced phenotypical changes. **(A)** DCs viability was assessed by trypan blue staining after collagenase treatment to disassemble the collagen scaffold (before centrifugation). **(B)** For flow cytometry analysis, cells were gated based on size, single cells, and live cells. Depicted is the HLA-DR-based gating strategy to distinguish PDTOs and DCs, using three conditions: DCs only, DCs and PDTOs co-culture, and PDTOs only. **(C)** Representative histogram plots to exemplify basal expression of CD86, HLA-DR, and PD-L1 markers in iDCs and mDCs. **(D)** Representative histogram plot of CD86, HLA-DR and PDL-1 to highlight the phenotypic shift of mDCs cultured in the presence of PDTOs. **(E)** Scattered dot plots showing normalized MFI values to iDCs and mDCs, respectively. Each dot/triangle represents a different donor, 4 donors were used in total. Data plotted as normalized values of raw MFI, mean with SD. The statistical significance between different conditions (mDCs/iDCs with and without PDTOs) was analyzed by a mixed-effects model followed by a Dunnett’s *post-hoc* multiple comparisons test on the log2 transformed ratio values. The statistical significance was annotated as follows: *p < 0.05, **p < 0.01. (Raw data can be found in **Supplementary Figure 5**).

After investigating cell interactions and the structure of the co-culture, we next evaluated if it was possible to retrieve the DCs from the collagen scaffold and assess the tumor organoids’ influence on their phenotype. To retrieve the cells, the collagen matrix was disassembled with collagenase. For assessment of DCs viability after 48h culture and collagenase treatment, the samples containing only mDCs or iDCs were stained with trypan blue prior to any centrifugation step. Results show that the viability was moderate to high for all DCs, albeit slightly lower for mDCs as compared to iDCs (**Figure 4A**).

Successful recovery of cells from the collagen matrix allowed surface stainings to be performed for immunophenotyping with flow cytometry, using HLA-DR expression to identify DCs (**Figure 4B**). Subsequently, we analyzed the phenotypic profile of DCs after being in contact with CRC organoids. For that, we analyzed the expression of the CD86 co-stimulatory molecule, the HLA-DR antigen presentation machinery, and the co-inhibitory molecule PD-L1 – all required for successful antigen presentation and priming of T cells. Representative histogram plots of CD86, HLA-DR, and PD-L1 highlight the distinct basal expression of the selected maturation markers in mDCs versus iDCs, confirming their phenotypic differences (**Figure 4C**). It is also shown in **Figure 4D**, as an example, a whole-population tumor-induced reduction in CD86 and HLA-DR expression in mDCs after 48h of co-culture with PDTO dense. In **Figure 4E** (and **Supplementary Figure 5**) it is shown how the expression of the markers is altered in iDCs and mDCs upon co-culture with PDTOs.

Interestingly, the results show that there is a decreased expression of the co-stimulatory CD86 marker in both iDCs and mDCs in the presence of the PDTOs (**Figure 4E**), suggesting a tumor-induced immunosuppressive effect. For HLA-DR, its expression remained stable in iDCs, whereas a decrease is noted for mDCs. PD-L1 expression was also impacted, and differently, by the two different PDTOs, but no statistically significant differences were observed. In general, co-culture with tumor cells seems to have a stronger impact on the expression of the studied markers on mDCs. And, notably, our system allows us to detect phenotypic differences induced by individual PDTOs; i.e., our PDTO dense seems to have a stronger suppressive effect on DCs than our PDTO cystic.

Together, these data demonstrate that after co-culture it is possible to retrieve DCs from the collagen matrix with high viability, which further allows phenotypic profiling and study of the impact of tumor organoids on DCs phenotype.

### 3.5 Functional analysis of DC activity following 3D co-culture

**Figure 5:**
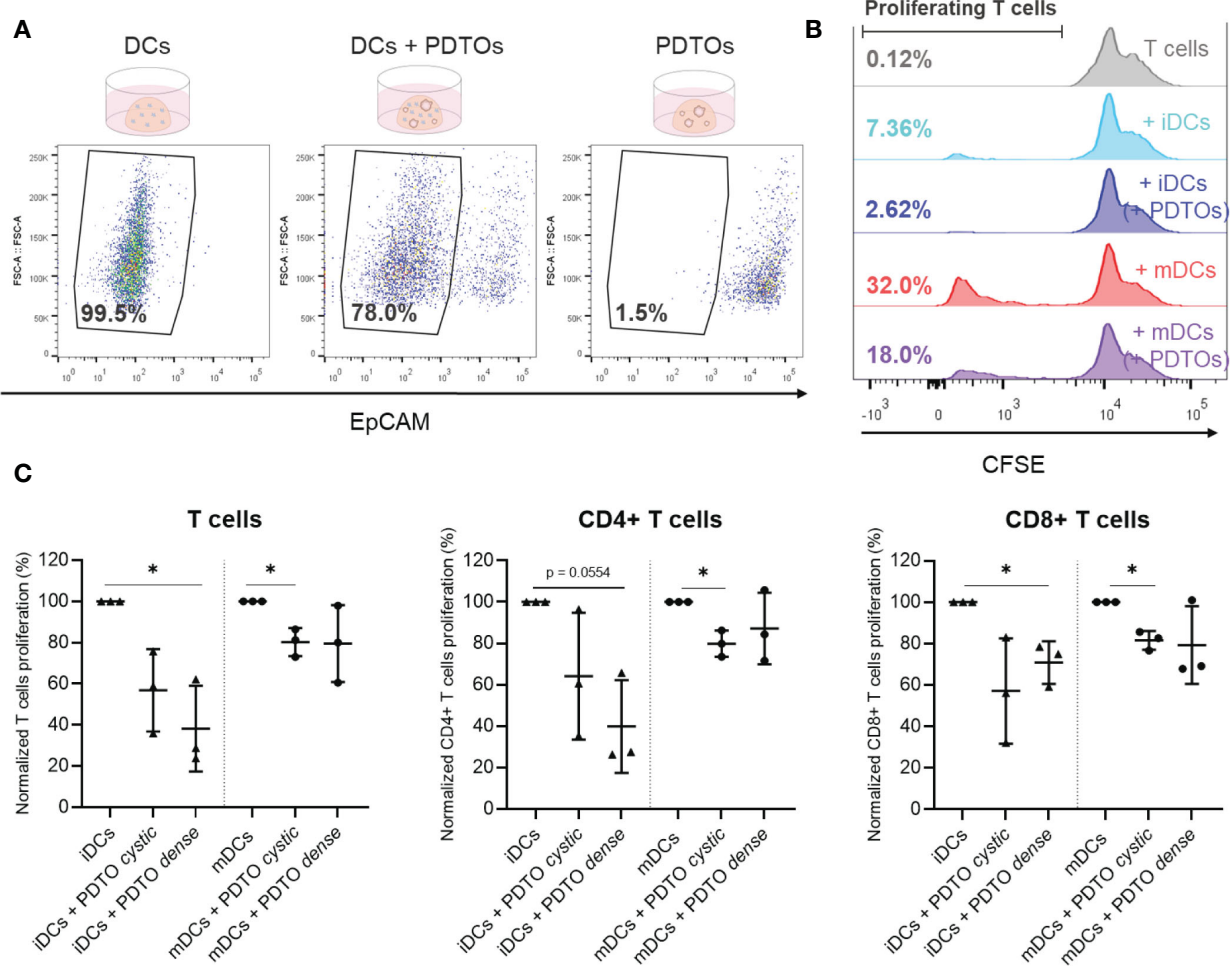
Sorting and functional characterization of DCs after co-culture with PDTOs – Allogeneic T cell assay. **(A)** Isolation of DCs, using EpCAM to sort out PDTOs. DCs gate defined based on EpCAM expression. **(B)** Representative CFSE histogram plots are shown. Numbers indicate the percentage of gated proliferating T cells. **(C)** Proliferation of allogeneic T cells after 6 days of co-culture with sorted DCs. Scattered dot plots show the percentage of proliferating T cells in each condition (average of technical replicates), normalized to proliferating T cells in the conditions with only iDCs and mDCs, respectively, mean with SD. The statistical significance between different conditions (mDCs/iDCs with and without PDTOs) was analyzed by a mixed-effects model followed by a Dunnett’s *post-hoc* multiple comparisons test on the log2 transformed ratio values. The statistical significance was annotated as follows: *p < 0.05. (Raw data can be found in **Supplementary Figure 6**).

Finally, we explored the possibility of functionally characterizing DCs after co-culture with the tumor organoids using an allogenic T cell assay based on HLA-DR mismatch, which provides information about the DCs’ ability to activate T cells and induce T cell proliferation. For this assay, a pure population of DCs is required after co-culturing. We therefore isolated DCs by FACS sorting out PDTOs with EpCAM labeling. Part of the used gating strategy is depicted in **Figure 5A**, demonstrating that EpCAM expression presents two clearly distinct populations (negative and positive). Representative histograms are presented in **Figure 5B**, where it is shown that iDCs have inherently a lower ability to activate T cell proliferation when compared to mDCs, as expected. Interestingly, we observed that DCs previously co-cultured with PDTOs were less capable of stimulating allogeneic T cells (both CD4+ and CD8+ T cells) proliferation when compared with iDCs and mDCs cultured alone (**Figures 5B, C** and **Supplementary Figure 5**).

Altogether, these results reveal that it is possible to perform functional readouts with DCs isolated from the 3D co-culture. Our results suggest that co-culture with PDTOs not only impacts DC phenotype but also their T cell activating abilities.

## 4 Discussion

Currently, there is a shortage of representative and dynamic *in vitro* models to study and dissect interactions between CRC and immune cells. In view of this gap, our main aim was to establish a 3D patient-derived co-culture model to mimic and investigate the interactions between DCs and metastatic CRC. Here, we present a co-culture of MoDCs and PDTOs in a 3D collagen matrix - amenable to live-cell microscopy, histological analysis, immunofluorescence, flow cytometry, and cell sorting - allowing comprehensive analysis and characterization of the impact of tumor cells on DCs phenotype and functions. As far as we know, this is the first study and model investigating human DCs in a tumor organoid 3D context.

### 4.1 Co-culture setup, cell viability and interactions, and structure

One of the main strengths of this study is the use of PDTOs as a tumor model. Firstly, several mechanisms are specific to humans and difficult to reproduce in animal models (35). Secondly, the 3D architecture of the tumor organoids recapitulates and preserves both histological and mutational features of the original tumor, which is particularly relevant given the heterogeneity of CRC. Thirdly, PDTOs provide a high degree of translational information supporting their clinical relevance (36–38). Finally, previous studies show that patient-derived tumor organoid co-cultures with T cells recapitulate and preserve tumor-immune cell interactions and treatment response within the TME (27, 29).

Despite their physiological relevance, using only two organoids is a limitation of this study, which in the future can be surpassed by the use of additional organoid lines. MoDCs were chosen as a DC model, as these are the most used and accessible source of human DCs. Nevertheless, MoDCs are generated *ex vivo* from monocytes, as such, the use of primary DC subsets isolated directly from the blood would be desirable to further improve the physiological relevance of the system.

Another key point of the presented co-culture system is the 3D collagen matrix setup. The 3D environment allows spatiotemporal analysis and insights into tumor-DC dynamics. The collagen type and concentration were chosen based on previous work showing that it supports and allows DCs to migrate, locate and engage with each other and with other cells in co-culture (32, 39). Key challenges of 3D co-culture systems include the batch-to-batch variability of scaffold materials, costs, and the absence of important elements such as vascular flow or interaction with other organs (26, 40). Some of these challenges can be partially overcome in more complex microphysiological chip platforms as previously described (31). Nevertheless, we believe that the here proposed simple, feasible, and reproducible setup and toolbox is valuable to examine 1-1 immune cell-tumor interactions and mediators while maintaining tumor heterogeneity, spatiotemporal interactions, and physiological relevance.

In line with previous research, in our co-culture model, the collagen setup and concentration support cell viability and 3D disposition of both DCs and PDTOs. Moreover, it fosters DC functions, including migration and engulfing of tumor fragments, and facilitates DC-tumor interactions. As described in our study, DCs - in particular, iDCs likely due to their increased phagocytic ability in comparison with mDCs (41) - cluster around and inside the tumor organoids, sampling tumor material, and extensively interact with the organoids. This was the first milestone to be achieved, as the goal of this system was to be able to study and uncover DC-tumor interactions.

Next, we wanted to compare the structure and cell organization within our co-culture with patient tissue sections. Interestingly, we found the distribution of DCs within the co-culture system to be maturation status-dependent, and different for both PDTOs in the study, potentially showing the adaptability and specificity of the model. Remarkably, our relatively simple co-culture system, is comparable to mCRC patient sections, in terms of tumor lesion morphology, DC distribution, and distance range to tumor lesions for the samples analyzed. Of note, the patient tissue sections were not from the same patients from which the organoids were derived, further suggesting the representability of the system.

### 4.2 Tumor-induced DC phenotype and (dys)function

Tumor-infiltrating DCs are known to perform crucial functions, such as reinvigorating, activating, and modulating the magnitude and duration of T cell responses, and recruiting and regulating effector T cells influx to the tumor site. These functions are crucial not only for the coordination of anti-tumor T cell responses, but also for immunotherapy effectiveness. DCs achieve this by either the generation of chemokine/cytokine gradients or direct antigen presentation (11, 12, 42, 43). MoDCs can prime Th1 and cytotoxic immune responses and have been shown to play an important role in different physiological and inflammatory settings including tumors (44–47). In our co-culture system, we include and study immature and mature MoDCs, as both functional states can be found within the TME and, greatly influence the quality and pro- or anti-tumor direction of immune responses (12).

Retrieving viable DCs from the 3D co-culture system was crucial for studying tumor-induced phenotypic and functional changes. Our results corroborate the different behaviors, phenotypes, and functions of mDCs and iDCs. For instance, in an immature state DCs are more active in tumor engulfing, whereas in a mature state they are specialized in antigen presentation and T cell activation. Overall, our results suggest that the expression of co-stimulatory molecules (CD86), antigen presentation machinery (HLA-DR), and co-inhibitory molecules (PD-L1) in DCs, and their ability to activate T cells were impacted upon interaction with tumor organoids. This suggests that the tumor shifted or locked DCs in a more immature state, associated with tolerance and pro-tumorigenic effects. Future research, building on our model, can further characterize the functional consequences of tumor-induced DC dysfunction on T cell biology and dissect associated mediators and mechanisms

The observed phenotype shift with impaired maturation and T cell activation abilities is in line with previous studies investigating DCs phenotype and function in patients, and also in a study assessing the impact of tumor-derived supernatant on DC maturation (48–50). We observed a stronger tumor impact on mDCs than on iDC phenotype, this might be related to a higher basal expression of the studied markers on mDCs or perhaps due to a higher sensitivity to environmental cues. Importantly, a stronger effect on mDCs would benefit the tumor since the presence of impaired mDCs has a stronger repercussion on mounting effective anti-tumor responses.

We also found that the two PDTOs used had different impacts on iDCs and mDCs behavior, distribution, recruitment, activation, and function. Notably, our data demonstrate that DCs interacted less with and were not in as close proximity (or inside) to PDTO dense when compared with PDTO cystic. Paradoxically, it was PDTO dense that had a more pronounced effect on DC phenotype. We speculate that this PDTO’s stronger immunosuppressive effect may be related to DC exclusion from the tumor surroundings. Further research including PDTO secretome profiles would be required to test this hypothesis.

Of note, the observation of morphologically distinct glandular structures, i.e., cystic versus dense, or ‘solid’—that characterize CRCs and are frequently recapitulated in tumour organoids, stems from the first CRC organoid biobank reported (51). However, our methods-focused exploratory study cannot make any claim ascribing functional differences to these two phenotypes based on single representatives. Additional experiments are needed to confirm a biological difference between any type of feature - including, e.g., mutational status - that these PDTOs can represent. Nevertheless, our results do suggest that our model may be sufficiently robust and feasible for the careful classification of CRC-DC interactions using larger numbers of organoids, or even for the assessment of patient-specific tumor-induced DC dysfunction.

Altogether, these findings support the physiological relevance of the tumor-mediated effects observed within our 3D co-culture system, indicating that the presented tool is a valuable additional approach to studying DC–CRC interactions. This model allows real-time investigation of tumor organoids modulating DCs phenotype and behavior. Importantly, getting insight into how CRC shapes DC maturation and functionality paves the way for the development of new therapies to prevent tumor-induced DC dysfunction, or restore their full anti-tumor potential. And hence, getting one step closer to promoting tumor destruction, avoiding metastasis formation, or unleashing treatment response in patients.

### 4.3 Future perspectives

Our study raises several additional opportunities for future research, and we view it as a promising starting point and a toolbox to be exploited and adapted for more complex and detailed studies of CRC-DC interactions within the TME. In the future, we believe that the presented co-culture system can be exploited for studies with primary DCs subsets and different tumor organoids. This can aid our understanding of (1) the individual contributions of the different DC subsets - with inherently different functional specializations; (2) and the tumor-specific mechanisms and mediators that regulate the fate of DC subset-mediated anti-tumor responses or tolerance within the TME. Furthermore, this knowledge might open doors for the (3) identification of potential targets and biomarkers for the design of DC subset-specific interventions. Finally, (4) possibly this system could be used for patient/organoid-specific studies if the physiological relevance and predictive power of the co-culture are confirmed by correlating *in vitro* outcomes with patients’ parameters such as tumor T cell infiltration or response to immunotherapy. Potentially, this approach can bring us closer to making existing or new immunotherapies available for more mCRC patients.

## Data availability statement

The raw data supporting the conclusions of this article will be made available by the authors, without undue reservation.

## Ethics statement

The studies involving human participants were reviewed and approved by ORCHESTRA trial (NCT01792934). The patients/participants provided their written informed consent to participate in this study.

## Author contributions

BS: conceived, performed the experiments, analysed the data, and wrote the manuscript. KI, DP, LB, HV: established and provided essential biological material. MG: assisted with immunohistochemistry experiments in patient samples. JC/KD/AC: assisted with experiment setup, data analysis, and interpretation. IV and DT: supervised the entire project. All authorscontributed to the article and approved the submitted version.
