# Supplementary material for: Dendritic cell phenotype and function in a 3D co-culture model of patient-derived metastatic colorectal cancer organoids
Source: Front Immunol. 2023 Jan 25;14:1105244. doi: 10.3389/fimmu.2023.1105244 (PMC9905679; doi:10.3389/fimmu.2023.1105244)
Supplement: Supplementary file 1 [file DataSheet_1.docx]

Supplementary Material

# Supplementary Data

Movie 1. Interactions between iDCs and PDTO dense.

Movie 2. Interactions between iDCs and PDTO cystic.

# Supplementary Figures


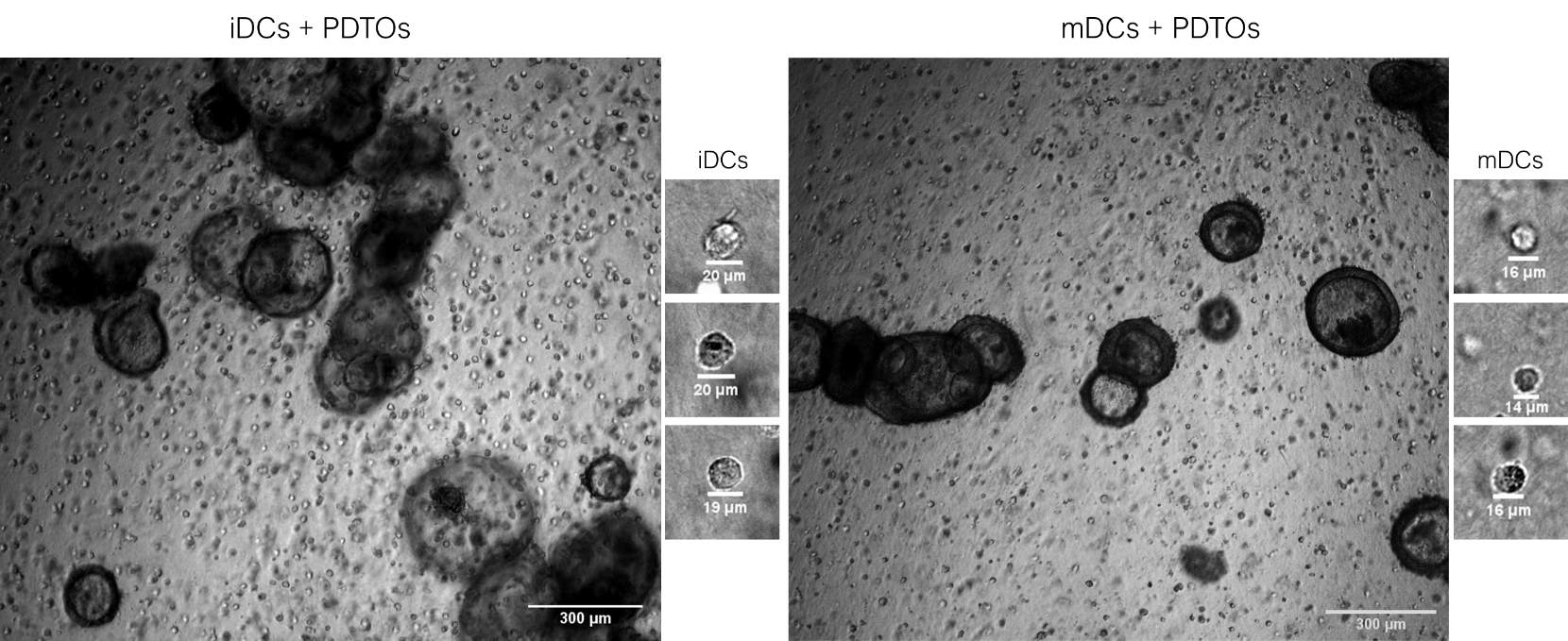


Supplementary Figure 1. Overview of co-cultures: iDCs and mDCs + PDTOs. Brightfield images, (5x magnification) displaying the cells’ distribution within the collagen, and size differences between iDCs and mDCs.


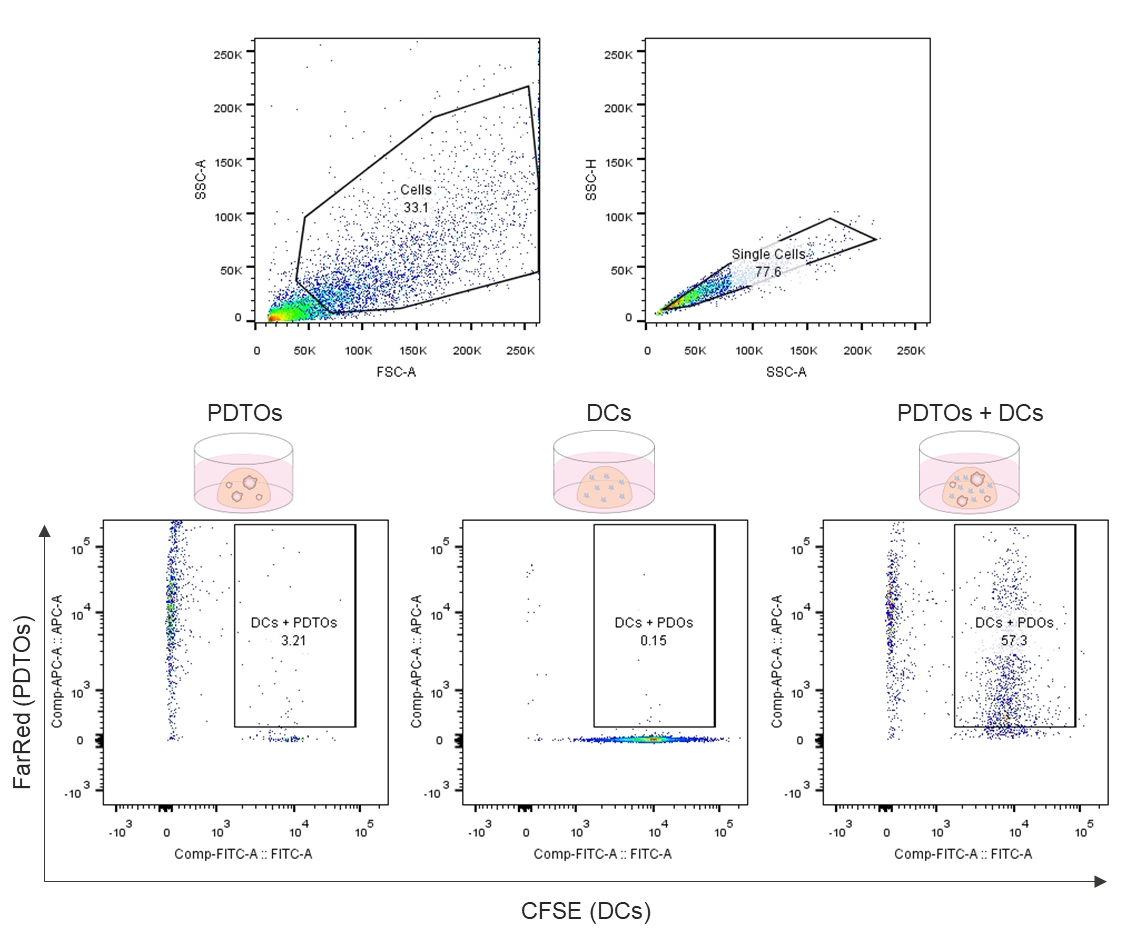


Supplementary Figure 2. Flow cytometry-based tumor cell uptake assay. Prior to co-culture iDCs and PDTOs were labelled with fluorescent dyes, CFSE (FITC channel) and FarRed (APC channel), respectively. After co-culture, a new population arises (57.3%), positive for both markers, this population is virtually absent when PDTOs (3.21%) and iDCs (0.15%) are cultured alone. This suggests engulfment/uptake of tumor cells by iDCs within the co-culture.


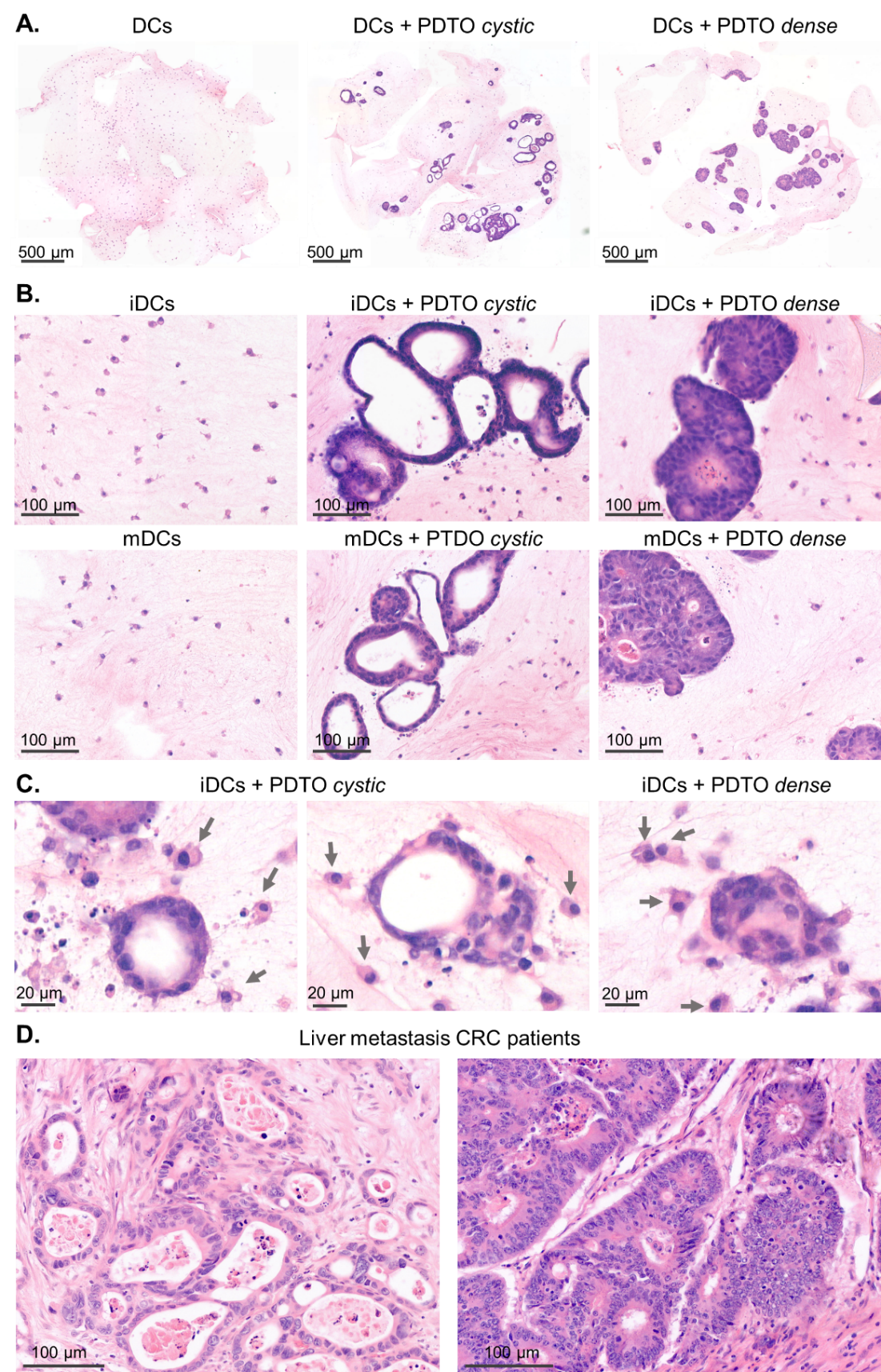


**Supplementary Figure 3.** **H&E stainings visualizing the structure and organization of DCs and PDTOs co-culture and patient sections of CRC liver metastasis.** **(A)** Overview of co-culture structure and organization. **(B)** Distribution of DCs in relation to the tumor organoids. **(C)** Interactions between DCs and PDTOs (examples pinpointed by arrows). **(D)** In patient sections of CRC liver metastasis, the left section resembles the cystic nature of PDTO cystic, and the right section is more comparable to the compact morphology of PDTO dense. Legend: iDCs - immature MoDCs, mDCs - mature MoDCs, PDTOs - patient-derived tumor organoids.


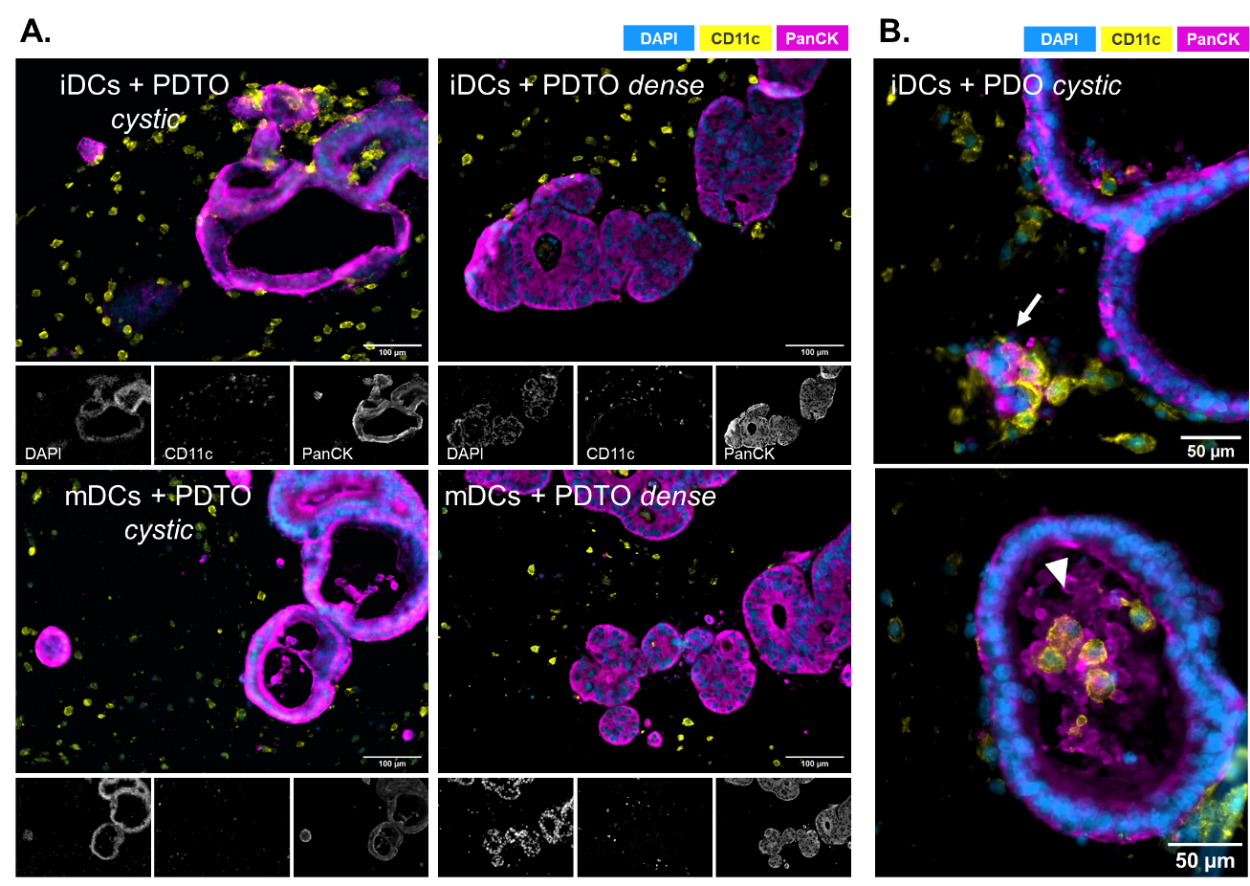


**Supplementary Figure 4.** **DCs distribution in relation to the tumor organoids within the co-culture. (A)** Immunofluorescence stainings of CD11c and PanCK in fixed sections of co-cultures. DCs are present surrounding the tumor organoids. Scale bar 100 µm. **(B)** Closeup of iDCs surrounding tumor-derived fragments, and inside and surrounding the tumor organoids. DCs cluster around and inside PDTOs (examples pinpointed by arrows).

**Supplementary Figure 5.** **Phenotypic characterization of DCs after 48h co-culture with tumor PDTOs.** Scattered dot plots showing raw MFI values, highlighting the differences in phenotype between iDCs and mDCs. Each dot/triangle and color represents a different donor, at least 4 donors were used for each condition. Data plotted as raw MFI, mean with SD.

**Supplementary Figure 6.** **Sorting and functional characterization of DCs after co-culture with PDTOs – Allogeneic T cell assay.** Proliferation of allogeneic T cells after 6 days of co-culture with sorted DCs. Scattered dot plots show the percentage of proliferating T cells in each condition (average of technical replicates) mean with SD, highlighting the increased ability of mDCs to induce T cell proliferation in comparison to iDCs. Each color represents a different donor.
